# *Gallibacterium anatis* infection in poultry: a comprehensive review

**DOI:** 10.1007/s11250-023-03796-w

**Published:** 2023-10-27

**Authors:** Wafaa A. Abd El-Ghany, Abdelazeem M. Algammal, Helal F. Hetta, Ahmed R. Elbestawy

**Affiliations:** 1 Poultry Diseases Department, Faculty of Veterinary Medicine, Cairo University, Giza, 12211 Egypt; 2 Bacteriology, Immunology, and Mycology Department, Faculty of Veterinary Medicine, Suez Canal University, Ismailia, 41522 Egypt; 3 Medical Microbiology and Immunology Department, Faculty of Medicine, Assiut University, Assiut, 71515 Egypt; 4 Poultry and Fish Diseases Department, Faculty of Veterinary Medicine, Damanhour University, El-Beheira, 22511 Egypt

**Keywords:** *G. anatis*, Virulence factors, Pathogenesis, Diagnosis, Antimicrobial sensitivity, Vaccines

## Abstract

*Gallibacterium anatis* (*G. anatis*), a member of the *Pasteurellaceae* family, normally inhabits the upper respiratory and lower genital tracts of poultry. However, under certain circumstances of immunosuppression, co-infection (especially with *Escherichia coli* or *Mycoplasma*), or various stressors, *G. anatis* caused respiratory, reproductive, and systemic diseases. Infection with *G. anatis* has emerged in different countries worldwide. The bacterium affects mainly chickens; however, other species of domestic and wild birds may get infected. Horizontal, vertical, and venereal routes of *G. anatis* infection have been reported. The pathogenicity of *G. anatis* is principally related to the presence of some essential virulence factors such as *Gallibacterium* toxin A, fimbriae, haemagglutinin, outer membrane vesicles, capsule, biofilms, and protease. The clinical picture of *G. anatis* infection is mainly represented as tracheitis, oophoritis, salpingitis, and peritonitis, while other lesions may be noted in cases of concomitant infection. Control of such infection depends mainly on applying biosecurity measures and vaccination. The antimicrobial sensitivity test is necessary for the correct treatment of *G. anatis*. However, the development of multiple drug resistance is common. This review article sheds light on *G. anatis* regarding history, susceptibility, dissemination, virulence factors, pathogenesis, clinical picture, diagnosis, and control measures.

## Introduction

Certain infections of poultry have a tremendous direct adverse impact on egg production or an indirect effect on the health status of poultry. One of these bacterial infections is *Gallibacterium anatis* (*G. anatis*) infection. *G. anatis* is a member of the family *Pasteurellaceae* (Christensen et al. 2003; Bisgaard et al. 2009). In 1981, the separate taxonomy of the family *Pasteurellaceae*, which mainly consisted of isolated Gram-negative coccobacilli from animals, was established. To date, this family includes 15 genera: *Pasteurella*, *Avibacterium*, *Actinobacillus*, *Gallibacterium*, *Haemophilus*, *Mannheimia*, *Aggregatibacter*, *Bibersteinia*, *Lonepinella*, *Phocoenobacter*, *Histophilus*, *Nicoletella*, *Volucribacter*, *Chelonobacter*, and *Basfia* (Janda 2011)*.*

According to several studies, *Gallibacterium* caused deaths in domestic birds and occasionally people, suggesting it was more dangerous than an opportunistic infection (Driessche et al. 2020). The importance of *G. anatis* was underestimated for a long time due to an incomplete understanding of the pathogenesis, virulence, and growth kinetics (Kristensen et al. 2011). However, the overall rate of *G. anatis* disease increased in layer, breeder, and boiler chicken flocks in previous years (Elbestawy et al. 2018; Krishnegowda et al. 2020; Algammal et al. 2022a). These days, this newly developing illness poses a grave threat to the world’s chicken meat and egg industry (Antenucci et al. 2020; Algammal et al. 2021a; Elewa, 2021; Sorescu et al. 2021). The disease is widely distributed in American, European, Australian, African, and Asian countries. Although *G. anatis* may be regarded as a normal component of the microbiota of the lower genital, terminal digestive, and upper respiratory tracts under certain environmental and stress circumstances, it initiates reproductive and systemic disorders (Paudel et al. 2015; Ataei et al. 2017). Domestic and non-domestic species of birds and mammals, including humans, are susceptible to *G. anatis* infection (Aubin et al. 2013; Krishnegowda et al. 2020).

This bacterium has several virulence factors causing reproductive and respiratory tissue damage, particularly in the presence of co-infections and other stressors related to environmental conditions (Paudel et al. 2017a,b). *G. anatis* infection causes either local or systemic affection and is typically accompanied by a notable decline in laying performance, 5–10% egg drop, adverse changes in the eggshell quality, increased mortality, respiratory manifestations, and diarrhea (Paudel et al. 2014a). Oophoritis, salpingitis, epididymitis, peritonitis, septicemia, respiratory tract lesions, enteritis, hepatic necrosis, and pericarditis are the most common lesions of *G. anatis* infections (Driessche et al. 2020; Krishnegowda et al. 2020).

Recent and advanced diagnostic and identification techniques were adopted alongside routine cultural or biochemical phenotypic assays and molecular characterization for rapidly detecting *G. anatis* infection (Christensen et al. 2003; Huangfu et al. 2012).

Despite the sensitivity of *G. anatis* to many antimicrobials, some cases are non-responsive, and disease recurrences were reported. The development of multi-drug resistance (MDR), notable antigenic variation, and ineffective elimination of *G. anatis* by the host are the key limitations for disease control (El-Adawy et al. 2018; Hess et al. 2019). The widespread antibiotic resistance leads to ineffective treatment (Johnson et al. 2013). The antibiotic sensitivity of *G. anatis* strains continuously varies; thus, frequent in vitro assessment of strains is essential (Elbestawy et al. 2018). Few vaccines were exploited against *G. anatis* and are being estimated. To better understand *G. anatis* infection in poultry, this review discusses *G. anatis* history, susceptibility, dissemination, virulence factors, pathogenesis, clinical picture, diagnosis, and control and preventive measures.

## History

In 1950, *G. anatis* was found as a component of normal microbiota in the cloacae of apparently healthy chickens and designated as a “hemolytic cloaca bacterium” (Kjos-Hansen 1950). Later on, DNA hybridization demonstrated that avian *Pasteurella hemolytica* (*P. hemolytica*), *Actinobacillus salpingitidis* (*A. salpingitidis*), and *P. anatis* belong to different genera inside the family *Pasteurellaceae* (Bisgaard 1977), while Christensen et al. (2003) classified *Gallibacterium* into a separate and independent genus. Taxon 1, the 3rd group of strains labeled *P. anatis*, was closely related to *A. salpingitidis* and avian *P. haemolytica* **(**El-Adawy et al. 2018). *P. hemolytica* was then re-classified within the family *Pasteurellaceae* into *G. anatis* biovar hemolytica based on *16S rRNA* gene sequences (Christensen et al. 2003; Bojesen et al. 2008; Bisgaard et al. 2009). Now, the genus consists of 4 known species [*G. anatis* (biovar haemolytica and biovar anatis), *G. salpingitidis*, *G. melopsittaci*, and *G. trehalosi fermentans*], 3 genomospecies (1, 2, and 3), and unnamed group V (Christensen et al. 2003; Bisgaard et al. 2009; Janda 2011). *G. anatis* is a Gram-negative pleomorphic, capsulated, non-motile, non-spore former, and facultative anaerobic bacterium [requires micro-aerophilic conditions for growth on blood agar medium supplemented with 5–10% carbon dioxide (CO_2_)] (Christensen et al. 2003). According to Bisgaard (1982), phenotypically, *G. anatis* is divided into the “hemolytica” biovar, which produces β-hemolysis, and the “anatis” biovar, which is not a hemolytic variation.

The disease emerged in several continents, such as Europe, the Americas, Australia, Africa, and Asia, indicating these bacteria’s importance and worldwide distribution. For instance, *G. anatis* infections have been reported in Europe, including Germany (Matthes et al. 1969; Matthes and Loliger 1976; Mraz et al. 1976; Bisgaard 1977), Denmark (Bisgaard 1977; Bojesen 2003), Austria (Mirle et al. 1991; Neubauer et al. 2007), England, Norway, Sweden, and Czech Republic (Jordan et al. 2005; Galaz-Luna et al. 2009), and Romania (Sorescu et al. 2021). Moreover, in American countries, i.e., the USA (Shaw et al. 1990; Zellner et al. 2004; Jones et al. 2013), Canada (Shapiro et al. 2013), Mexico (Vazquez et al. 2003; Bojesen et al. 2007a, 2008; Chavez et al. 2017b), and Peru (Mendoza et al. 2014), cases of *G. anatis* were recorded. In addition, *Gallibacterium* have been recorded in Australia (Gilchrist, 1963), some African countries, such as Nigeria (Addo and Mohan, 1985; Lawal et al. 2017), Egypt (Elbestawy, 2014; Sorour et al. 2015; Abd El-Hamid et al. 2016, 2018; Mataried, 2016; Elbestawy et al. 2018; Elewa, 2021; Algammal et al. 2022a), and Morocco (Nassik et al. 2019), and many Asian counties, i.e., Japan (Suzuki et al. 1996; Huangfu et al. 2012; Zhang et al. 2017), Taiwan (Lin et al. 2001), China (Chuan-qing et al. 2008; Guo, 2011), India (Singh, 2016; Singh et al. 2016, 2018), Iran (Ataei et al. 2017, 2019; Allahghadry et al. 2021), Turkey (Yaman and Sahan 2019), and Syria (Janetschke and Risk 1970). The incidence of *G. anatis* infection in various parts of the world is depicted in Table 1.

**Table 1:** The first detection, incidence rate, and diagnostic methods of *G. anatis* infection worldwide (after establishment of new taxonomy, Gallibacterium, in 2003)

Continent	Country	Type of birds	Collected organs	Diagnostic method(s)	Incidence rate (%)	Reference(s)
Africa	Egypt	Layer chickens	Trachea, ovary, and oviduct	Phenotypic identification, cPCR, and sequencing	13.1	Elbestawy (2014)
	Nigeria	Muscovy ducks	Tracheal and cloaca swabs and ovarian tissue	Phenotypic identification	33.2	Lawal et al. (2017)
	Morocco	Layer chickens	Ovary, trachea, and cloaca	Phenotypic identification and cPCR	100	Nassik et al. (2019)
Europe	Denmark	Layer and breeder chickens	Trachea and cloaca	Phenotypic identification and cPCR	28.8	Christensen et al. (2003) Bojesen et al. (2003a, b)
	Austria	layer chickens	Oviduct	Phenotypic identification	96.7	Neubauer et al. (2007)
	Poland	Peacocks	Lung	Phenotypic identification	5	Rzewuska et al. (2007)
	Romania	Backyard chicken flock	Ovaries, lung, heart, and spleen	Phenotypic identification	3.3	Sorescu et al. (2021)
Asia	China	Layer chickens	Salpinx, ovary, liver, spleen, and upper respiratory tract	Phenotypic identification and cPCR	100	Chuan-qing et al. (2008)
	India	Ducks, pigeons, broiler, and layer chickens	Heart blood, spleen, and tracheal swabs	Phenotypic identification and cPCR	100	Singh et al. (2018)
	Turkey	Layer chickens	Trachea, lung, liver, and heart	Phenotypic identification and cPCR	2.2	Yaman and Sahan (2019)
	Iran	Layer chickens	Ovaries, oviduct, uterus, cloaca, and abdominal cavity	Phenotypic identification and cPCR	NA	Ataei et al. (2017)
Australia	Australia	Layer chickens	Cloaca and oviduct	Phenotypic identification	55.5–100	Shini et al. (2013)
North America	USA	Layer chickens	NA	Phenotypic identification, cPCR, and sequencing	NA	Johnson et al. (2011)
		Broiler and breeder chickens	Joints, wattles, lungs, abdomen, and heart	Phenotypic identification, cPCR. and sequencing	NA	Jones et al. (2013)
		• Immature broiler breeder chickens • Breeder chickens • Broiler chickens • Layers hens	Liver, oviduct, ovary, joint, bone, lung, pericardium, and spleen	Phenotypic identification	NA	Shapiro et al. (2013)
	Mexico	Layer chickens	Lung, trachea, ovary	Phenotypic identification and cPCR	76.6	Castellanos et al. (2006) Chavez et al. (2017a)
	Panama	Breeder chickens	NA	Phenotypic identification and serology	NA	Calderon and Thomas (2009)
South America	Peru	NA	NA	Restriction endonuclease enzyme of 16S rRNA gene sequences	NA	

*NA* not available

## Susceptibility

Numerous domestic and wild avian species, including chickens, ducks, geese, turkeys, pigeons, pheasants, guinea fowls, budgerigars, peacocks, parrots, partridges, cattle egrets, web-footed galliformes, psittacine birds, and owls, may harbor *G. anatis* (Mushin et al. 1980; Bisgaard, 1982; Bojesen et al. 2003a; Zellner et al. 2004; Bojesen and Shivaprasad 2007; Christensen et al. 2007a; Rzewuska et al. 2007; Bojesen et al. 2008; Persson and Bojesen, 2015; Sorour et al. 2015; Singh, 2016; Singh et al. 2016, 2018). Infection was reported even in non-avian species, namely, cattle, horses, pigs, sheep, and rabbits (Kristensen et al. 2011; Lawal et al. 2017). Furthermore, some *G. anatis* infections in humans have been detected (Aubin et al. 2013; Driessche et al. 2020). Regarding age susceptibility, older research studies have reported that young birds are less susceptible to *G. anatis* than adults (Bisgaard 1977; Mushin et al. 1980), whereas Huangfu et al. (2012) informed an extreme rate of detection and isolation of *G. anatis* from chickens aged 5–6, 12, 18, and 55–58 weeks. However, *G. anatis* was isolated from broiler chickens (Abd El-Hamid et al. 2016).

## Infection and dissemination

The primary route of *G. anatis* infection is the horizontal one through the respiratory tract (Bisgaard 1977). Therefore, this bacterium is primarily prevalent in flocks with low biosecurity measures (Bojesen et al. 2003a). Isolation of *G. anatis* from the different body organs indicates the systemic circulation and spread of the bacterium from its natural habitat (Zepeda et al. 2010). Affection of the reproductive organs may be associated with ascending infections from the cloaca (Bojesen et al. 2003a; Neubauer et al. 2009). There is experimental evidence of vertical transmission in embryonated eggs through the trans-ovarian/oviduct route or the trans-eggshell route (Matthes and Hanschke 1977; Persson and Bojesen, 2015; Wang et al. 2018).

*G. anatis* was isolated from the egg yolk and ovaries of hens 10 days after the experimental infection (Shapiro et al. 2013; Paudel et al. 2014a). In embryonated eggs, *G. anatis* may penetrate the eggshell pores and infect the growing embryo causing mortality (Wang et al. 2018). Besides, venereal transmission has been suggested since *G. anatis* damages semen quality and causes epididymitis (Paudel et al. 2014b). Semen of *G. anatis* infected cockerels may function in transmission among adult birds and probably to their offspring.

Intranasal inoculation of cockerels with *G. anatis* resulted in the presence of the pathogen in the testis and epididymis within a week post-infection (pi). In addition, the sperm density was lower, total and progressive motility were lower, and membrane integrity was worse in the semen, all indicating a detrimental effect on fertility. The study of Neubauer et al. (2009) revealed that *G. anatis* affected 13-layer flocks, and the birds suffered from cannibalism (extensive wound pecking around the cloaca).

### Zoonotic importance

It is presumed that *G. anatis* is a food contaminant that transmits to humans. In 2013, in France, the first case of bacteremia initiated by *G. anatis* was reported in an immunocompromised woman infected through consuming contaminated food. The predominant symptoms include fever, severe abdominal pain, anemia, diarrhea, and neutropenia (Gautier et al. 2005; Aubin et al. 2013). Driessche et al. (2020) reported that poultry and cattle have a likely risk for zoonotic transmission of *G. anatis*, and further research should be conducted to establish their zoonotic potential. Recently, Wang et al. (2023) reported a case of acute watery diarrhea (7–8 times daily) brought on by *G. anatis* in a 62-year-old man with type 2 diabetes and hypertension. There was no urgency, no bloating or pain in the abdomen, no dread of a cold or fever, and only nausea and vomiting were the symptoms of the illness. The bacteria were identified using *16S rRNA* sequencing and matrix-assisted laser desorption/ionization time-of-flight mass spectrometry (MALDI-TOF–MS).

## Virulence factors

**Fig. 1:**
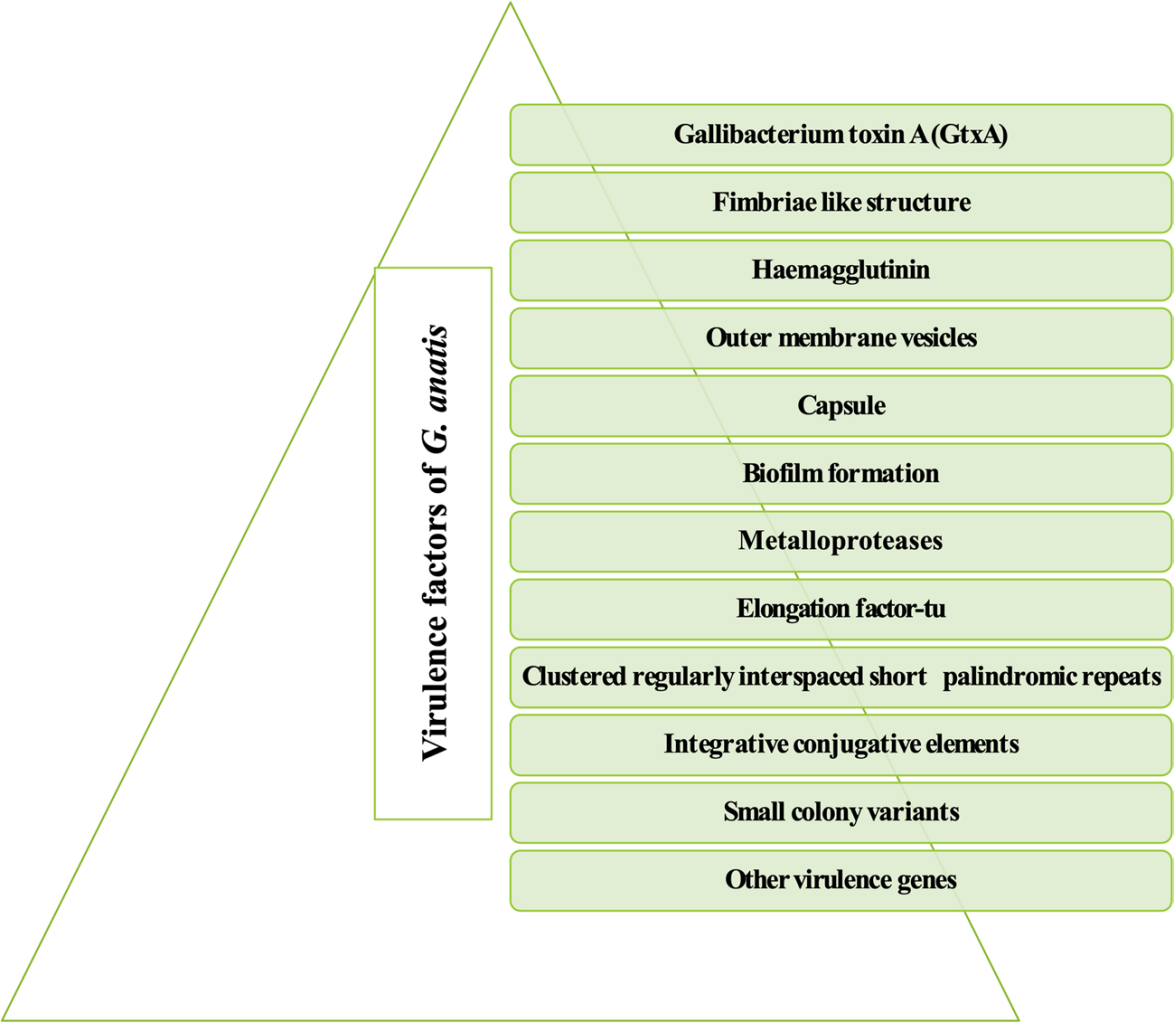
Virulence factors of *G. anatis* biovar haemolytica

The different virulence factors of *G. anatis* are illustrated in Fig. 1.

### *Gallibacterium *toxin A 

*Gallibacterium* toxin A (GtxA) is a vital virulence protein that is responsible for the hemolytic property of *G. anatis* biovar hemolytica (Kristensen et al. 2011). It has a leukotoxic impact on chicken macrophage cell line HD11 and lyses red blood cells (RBCs) (Kristensen et al. 2010; Persson and Bojesen 2015), and stimulates the immune response (Bager et al. 2014). This toxin has two domains, the C- and N-; both are needed for hemolytic activity. The C-terminus is similar to repeat in toxins (RTX) of the *Pasteurellaceae* family, while the N-terminal has RBCs lytic and leukocidal actions (Yang et al. 2020). The GtxA knockout mutant bacterium was exposed to a reduction of pathogenicity. It is comparable to the cytoskeletal protein talin, which binds integrins to the actin and vacuolar cytoskeleton and is crucial for interactions between the host and the bacteria (Kristensen et al. 2010). Actin can support cell motility, recognition, adhesion, phagocytosis, signal modulation of immune cells, and free radical generation. When GtxA binds to actin in host immune cells, the cell shape can be altered, and cellular signal transmission is impeded, and as a result, the bacteria can elude the host immune system (Aktories et al. 2011). Nassik et al. (2019) demonstrated that all the molecularly identified strains of *G. anatis* expressed the virulent GtxA. Tang et al. (2020) implied that GtxA plays a crucial function in an acute cytokine-mediated Th2-like response versus *G. anatis* infection in the ovarian tissue, helping in the pathogenesis of *G. anatis* infections in laying hens. Additionally, Tang and Bojesen (2020) attempted to explain the immunosuppressive effect of *G. anatis* GtxA during the interaction with chicken macrophage-like HD11 cells by encouraging cell adhesion and invasion, reducing the host inflammatory response based on an initial over-expression of interleukin (IL)-10 and a corresponding low-level expression of tumor necrotizing factor (TNF)-α, and concluded that GtxA induces cell death (apoptosis) without revealing clear causes. *Fimbriae.*

Strains of *G. anatis* can adhere to the host’s mucosal surface via hair-like structures, fimbriae, that belong to the F17-like family that contains 1–3 different fimbrial clusters (Johnson et al. 2013; Kudirkiene et al. 2014; Persson and Bojesen 2015). The F17-like fimbria (GalF-A) is encoded by 4 genes: *flf*D, *flf*C, *flf*G, and *flf*A (Bager et al. 2013b). These fimbriae can bind to N-acetyl-D-glucosamine receptors of the host (Klemm and Schembri, 2000; Vaca et al. 2011; Lucio et al. 2012; Kudirkiene et al. 2014), and the adhesion protein assists in interacting to receptors (Lintermans et al. 1991). In vivo, the fimbria protein (*flf*A) is crucial for virulence (Bager et al. 2013b). Accordingly, fimbrial expression can control *G. anatis* tissue tropism (Bager et al. 2013b). It has been found that *G. anatis* strains can adhere to chicken epithelial cells in vitro and inert surfaces via their short fimbria-like structures (Vaca et al. 2011; Lucio et al. 2012).

### Haemagglutinin

Some strains of *Gallibacterium* can agglutinate RBCS of avian and mammalian species (Zepeda et al. 2009; Ramirez-Apolinar et al. 2012). Though most strains of *G. anatis* can agglutinate rabbits’ RBCs, few strains may agglutinate the RBCs of chickens and quails due to the expression of haemagglutinins or adhesins binding receptors on the cells’ surface (Bager et al. 2013a; Johnson et al. 2013). Moreover, hemagglutinin protein was detected in biofilms and outer membrane vesicles (OMVs) liberated from *G. anatis* (Montes-Garcia et al. 2016).

### Outer membrane vesicles

Proteins, lipopolysaccharides, and DNA are found in the budding regions of the outer cell membrane, OMVs, of several Gram-negative bacteria (Mashburn-Warren and Whiteley 2006; Kulp and Kuehn 2010; MacDonald and Kuehn 2012). The enormous collection of core proteins found in the OMVs of *G. anatis* strains is mainly unaffected by the various in vitro growth conditions, but certain environmental stimuli greatly influence their expression. They facilitate bacterial adhesion and colonization, the formation of biofilms, and the removal of several antibiotic compounds (Bager et al. 2013a). Additionally, *G. anatis* releases hemagglutinin in the OMVs to agglutinate avian RBCs (Zepeda et al. 2009; Bager et al. 2013a, 2014; Johnson et al. 2013) and trigger a robust immunological response (Pors et al. 2016b).

### Capsule

Bojesen et al. (2007a) identified and characterized the genetic elements responsible for capsule biosynthesis in *Gallibacterium* for the first time. The capsule biosynthetic locus of *Gallibacterium* resembles the *Escherichia coli* (*E. coli*) group 2 capsule in structure and is considered a critical virulence factor for the pathogenesis of *G. anatis* infection. Some Gallibacterium strains have a polysaccharide capsule that adds virulence (Persson and Bojesen 2015). Capsule in *G. anatis* has an essential role in the adhesion and interaction of the bacterium with the surface of the host and immune evasion (Bojesen et al. 2011b; Singh et al. 2011; Harper et al. 2012). Although this capsule can be seen using electron microscopy (Bojesen et al. 2011c), it disappears following sub-cultures (Kjos-Hansen 1950). A capsule-knockout mutant (Δ*gex*D) became more virulent than its wild-type equivalent (Bojesen et al. 2011c).

### Biofilm formation

Biofilms mainly comprise proteins, polysaccharides, nucleic acids, and amyloid proteins (Costerton et al. 1999; Larsen et al. 2007; Lopez-Ochoa et al. 2017). Biofilm formation begins through the bacterium’s capacity to bind the cell’s inert surface. The adhesive capacity of *G. anatis* as a tool for colonization of tissue surfaces and for allowing infection to persist inside the host was studied by scanning electron microscopy, and the findings implied that all the used isolates had formed robust biofilms on polystyrene and glass within the first 3 h of exposure (Vaca et al. 2011). Strains of *G. anatis* are classified into 3 categories: weak, moderate, and strong groups according to the biofilm formation capability (Johnson et al. 2013). This may indicate that the development of biofilms is significant for particular bacterial clades. Additionally, the biofilm is crucial for the chronicity and duration of infections and for reducing their sensitivity to antibiotics (Costerton et al. 1999; Donlan and Costerton 2002; Persson and Bojesen 2015). It has been found that proteins in biofilms are capable of interacting with several host proteins, including fibronectin, fibrinogen, laminin, and plasminogen, and consequently alter the host's homeostasis (Epstein and Chapman 2008; Lopez-Ochoa et al. 2017).

### Metalloproteases

Metalloprotease enzymes are essential in *G. anatis* bacterium for proteolysis, increasing virulence, colonization, nutrient acquisition, and degradation of immunoglobulin (Ig) (G), along with bacterial invasion into the systemic circulation (Garcia-Gomez et al. 2005; Chavez et al. 2017b). It was found that these enzymes can down-regulate the immune response by acting on antibodies and complement system (Miyoshi and Shinoda 2000) that helps in colony establishment and bacterial transmission to blood circulation. In addition, proteases may be accountable for the host-specific pathogenicity of *G. anatis* strains (Zepeda et al. 2010). Metalloproteases such as metal-dependent endonuclease domain, zinc, and ATP-dependent metalloprotease are encoded in the *G. anatis* genome (Johnson et al. 2013).

### Elongation factor Tu

Elongation factor Tu is a protein released through vesicle formation, possesses amyloid characters, and is consequently included in the pathogenesis of *G. anatis* (Lopez-Ochoa et al. 2017).

### Clustered regularly interspaced short palindromic repeats (CRISPR)

According to Johnson et al. (2013), the bacterial innate defense system known as clustered regularly interspaced short palindromic repeats (CRISPR) breaks down invasive foreign and phage and plasmid nucleic acids. It can obstruct transformation, indicating that different strains of *G. anatis* have different levels of innate ability (Kristensen et al. 2012).

### Integrative conjugative elements

Integrative conjugative elements have specific distinguishing characteristics, such as a site-specific integrase, transfer genes, and genes that control excision and transfer. Genes encoded inside these elements have been used to identify them in the *G. anatis* genome (Wozniak et al. 2009; Johnson et al. 2013). They have genes for antibiotic resistance, which spreads resistance to other bacteria (Bojesen et al. 2011b).

### Small colony variants

Small colony variants are detected in the cultures of *G. anatis* as they have hemolytic activity (Greenham and Hill 1962). Moreover, they might aid in bacterial survival, recurrent infections, and the development of antibiotic resistance (Proctor et al. 2006).

### Other virulence genes

*G. anatis* pathogenicity has been linked to the genes *cps*16A, 16B, and 16F that encode the enzymes glycosyltransferase, hyaluronidase, and UDP-glucose 6-dehydrogenase, respectively (Bosse et al. 2017), through the cloning and characterization of the gene encoding *G. anatis fnr*G (a homolog of the global regulator gene conferring a hemolytic phenotype in *E. coli*) and its function in the production of the *E. coli* silent hemolysin. Bojesen (2003) investigated the relationship between *G. anatis* and *E. coli*. *she*A was studied by creating an *E. coli*-*she*A, a null mutant of the silent hemolysin, from a 4.2 kilobase pair hind segment harboring *fnr*G. The fact that *fnr*G activates *she*A and produces a non-hemolytic transformant led him to conclude that *fnr*G is probably a member of the *fnr* global regulatory protein family.

## Pathogenicity

The bacterium was isolated from apparent healthy chickens’ nasal and tracheal passages and cloaca as a part of their normal microbiome (Bojesen et al. 2003a). Several studies verified the existence of *G. anatis* as a key bacterial pathogen in both natural and experimental infections. The association of *G. anatis* with septicemia, oophoritis, salpingitis, egg abnormalities, pericarditis, hepatitis, tracheitis, and elevated mortality in pullets signifies that at least some strains of Gallibacterium possess pathogenic possibility in chickens (Bojesen et al. 2004; Neubauer et al. 2009; Jones et al. 2013; Paudel et al. 2013, 2014a, b, 2015; Elbestawy 2014; Persson and Bojesen 2015). The differentiation between pathogenic and non-pathogenic isolates of *G. anatis* using embryo lethality assay failed to obtain any significant results because both *G. anatis* isolates from healthy and sick chickens caused hemorrhagic lesions and death of embryos in 70 to 100% of inoculated eggs (Trampel and Nalon, 2008).

The virulence of the *G. anatis* strain, the infection’s route, and the immune status and age of the host are factors that influence and exacerbate the pathogenicity of the bacterium in chickens (Bisgaard, 1977; Bojesen et al. 2004, 2008). Concomitant infection with other viruses or bacteria (Matthes et al. 1969; Shaw et al. 1990), malnutrition, hormonal disturbance (Kohlert 1968; Gerlach, 1977), and environmental stressors such as seasonal variations (Mirle et al. 1991), cold (Matthes and Loliger, 1976; Rzewuska et al. 2007), inadequate ventilation, and overcrowding, as well as poor biosecurity, increase the severity of *G. anatis* infection (Verbrugghe et al. 2012; Paudel et al. 2015, 2017a, b; Persson and Bojesen, 2015). For instance, a co-infection of *G. anatis* with *E. coli*, *Avibacterium paragallinarum* (*A. paragallinarum*), and *Mycoplasma gallisepticum* causes increased morbidity and mortality in chickens (Neubauer et al. 2009; Paudel et al. 2017a, b; Abd El-Hamid et al. 2018). Furthermore, the systemic infection was worsened by a bacterial infection that included the infectious bronchitis virus (He-ping et al. 2012; Mataried, 2016).

Experimental infection of naturally immunocompromised layer chickens with *G. anatis* led to 8–10% lowered egg production and a 73% mortality rate (Jordan et al. 2005; Shapiro et al. 2013). Infection of layer hens with *G. anatis* induced hemorrhagic oophoritis and rupture of ovarian follicles (Neubauer et al. 2009; Jones et al. 2013; Paudel et al. 2014a), while infection in cockerels resulted in epididymitis, decreased semen quality, decreased sperm density, altered overall motility, and loss of membrane integrity (Paudel et al. 2014b). The disease affects broiler chickens on a systemic level (Zepeda et al. 2010; Paudel et al. 2013; Zhang et al. 2019). Generally, *G. anatis* biovar hemolytica caused septicemia, oophoritis, salpingitis, peritonitis, liver necrosis, perihepatitis, pericarditis, airsacculitis, tracheitis, and enteritis in infected chickens (Bojesen et al. 2004, 2007a; Neubauer et al. 2009; Paudel et al. 2013).

Despite the previous pathological findings of *G. anatis*, it was found that this pathogen may colonize the upper respiratory and reproductive tracts without initiating substantial signs or lesions (Paudel et al. 2013, 2014a, b). Many virulence factors enable *G. anatis* to invade, adhere, and colonize the host’s surface epithelium. After oropharyngeal and oviduct epithelial cells infection with *G. anatis*, the pathogen adheres firmly via the reaction between the adhesin and the host’s cell surface receptor, followed by rampant multiplication, colonization, and syntheses of virulence factors (Vaca et al. 2011; Lucio et al. 2012; Bager et al. 2013a,b). *G. anatis* F149T can express fimbriae responsible for mucosal attachment and colonization to the epithelium of the oropharynx (Lucio et al. 2012). The in vitro study of Zhang et al. (2017) showed that higher adhesion to primary chicken oviduct epithelial cells and increased generation of inflammatory cytokines have been observed in the highly pathogenic strains of *G. anatis* [IL-6, TNF-α, and interferon (IFN-c)], resulting in inflammation and tissue injury. Many other specific virulence factors can influence the pathogenicity of *G. anatis*, such as IgG-degrading proteases, biofilm, hemagglutinin, fimbriae, capsule, GtxA, OMVs, metalloproteases, elongation factor Tu, and clustered regulatory short palindromic repeats (Bojesen et al. 2004; Garcia-Gomez et al. 2005; Christensen et al. 2007b; Zepeda et al. 2009; Kristensen et al. 2010; Lopez-Ochoa et al. 2017).

The primary target for *G. anatis* colonization is the respiratory tract, where the bacterium persists for 4 weeks pi, then spreads to the reproductive organs such as ovaries, oviducts, or even testicles, causing drop in egg production, inflammatory lesions, and mortality, and these pictures support the claim for its systemic infection (Shaw et al. 1990; Neubauer et al. 2009; Paudel et al. 2013; Abd El-Hamid et al. 2016). Systemic infection with *G. anatis* may occur, resulting in septicemia, which sets off an inflammatory cascade in several organs that causes inflammation of the upper respiratory tract, oophoritis, follicular hemorrhage, rupture, and degeneration, salpingitis, peritonitis, pericarditis, and perihepatitis (Harbourne, 1962; Kohlert, 1968; Janetschke and Risk, 1970; Hacking and Pettit 1974; Bisgaard, 1977; Gerlach 1977; Addo and Mohan, 1985; Majid et al. 1986; Shaw et al. 1990; Mirle et al. 1991; Suzuki et al. 1996; Neubauer et al. 2009).

Three *G. anatis* isolates from Mexico, China, and Austria were evaluated for their differences in pathogenicity in a specific pathogen-free Lohmann layer chicken population collected from various geographic locations. The results indicated that the Mexican isolate had a slightly higher pathogenicity than the two other strains, suggesting a geographical pathogenicity difference (Paudel et al. 2013). Regarding the genetic variety of isolates of *G. anatis*, Bojesen et al. (2003b) obtained 114 *G. anatis* isolates from tracheal and cloacal swab samples of organic flocks, egg-producing flocks, and layer parent chicken flocks. The findings showed higher genetic similarity (more than 94%) in the organic flock isolates. On the contrary, the layer parent flock isolates were divided into two subclusters containing each tracheal or cloacal isolated, with similarity above 90%. This genetic diversity, indicating clonal lineages, may have altered to several spots inside the same bird, granting them additional capability to initiate a disease (Bojesen and Shivaprasad 2007; Johnson et al. 2008).

## Clinical manifestations

Infection with *G. anatis* is associated with multiple and variable clinical pictures but is often non-specific. In broilers, the pathogen causes respiratory manifestations in the form of nasal discharge, swelling and shaking of the head, cough, rales, dyspnea, diarrhea, and emaciation (Bojesen et al. 2008; El-Adawy et al. 2018; Elbestawy et al. 2018). In addition, *G. anatis* infection may induce a 3–18% reduction in egg production in layers (Jones et al. 2013), while in breeders, it causes an increasing mortality rate of 0.06–4.9% (Elbestawy et al. 2018). Mixed respiratory and reproductive disorders could be noticed in layers and breeder chicken flocks (Ataei et al. 2017; Chavez et al. 2017a; Abd El-Hamid et al. 2018). Elewa (2021) diagnosed *G. anatis* in layer chickens suffering from sneezing, rales, gasping, coughing, head swelling, and a 5–10% drop in egg production. Moreover, Bojesen et al. (2004) investigated the pathology of *G. anatis* virulent strain 12656–12 by intravenous or intraperitoneal inoculation in normal or immunosuppressed commercial brown laying chickens aged 15 weeks old. The author found that intravenously infected birds had severe septicemic lesions in both the normal and immunosuppressed birds. Mortality (73%) was recorded with severe depression and reluctance to move chickens. While Hui-min et al. (2009) reported the relationship between *G. anatis* and layer oviduct cysts, they did not indicate whether these lesions were co-associated with other viral diseases such as infectious bronchitis and low pathogenic avian influenza (LPAI-H9N2) or not. The clinical signs and gross lesions of 42 naturally infected layers with *G. anatis* were observed and recorded. Furthermore, a combined infection among *G. anatis* and other pathogens was also investigated. Hyperemia, swelling, and epithelium cell degeneration or necrosis were found in the mucosa of the respiratory tract and mucosa of the oviduct that presented many neutrophilic granulocytes and ovarian cysts. Most importantly, the severity of the clinical manifestations induced by *G. anatis* is exaggerated by co-infection with other pathogens, such as *A. paragallinarum* in chickens. Paudel et al. (2017a) observed that chickens infected with both *G. anatis* and *A. paragallinarum* had more severe nasal secretions and swelling of infraorbital sinuses than those with a single infection. In addition, in the presence of *E. coli* infection, *G. anatis* caused higher egg embryonic mortality than eggs with a single infection (Wang et al. 2018).

Under experimental infection conditions, *G. anatis* induced typical respiratory signs and weight losses in broiler chickens (Abd El-Hamid et al. 2018). However, layer chickens exhibited whitish diarrhea with a 66 to 47% drop in egg production (Paudel et al. 2014a). Specific pathogen-free cockerels showed no signs following experimental infection with *G. anatis*, but there was an alteration in the semen quality (Paudel et al. 2014b). Intranasal inoculation of *G anatis* showed an extensive and regular bacterial distribution in the respiratory and reproductive tract till 28 days pi (dpi) vs. one dpi in intravenously inoculated birds (Jones et al. 2013; Paudel et al. 2013).

## Post-mortem lesions

**Fig. 2:**
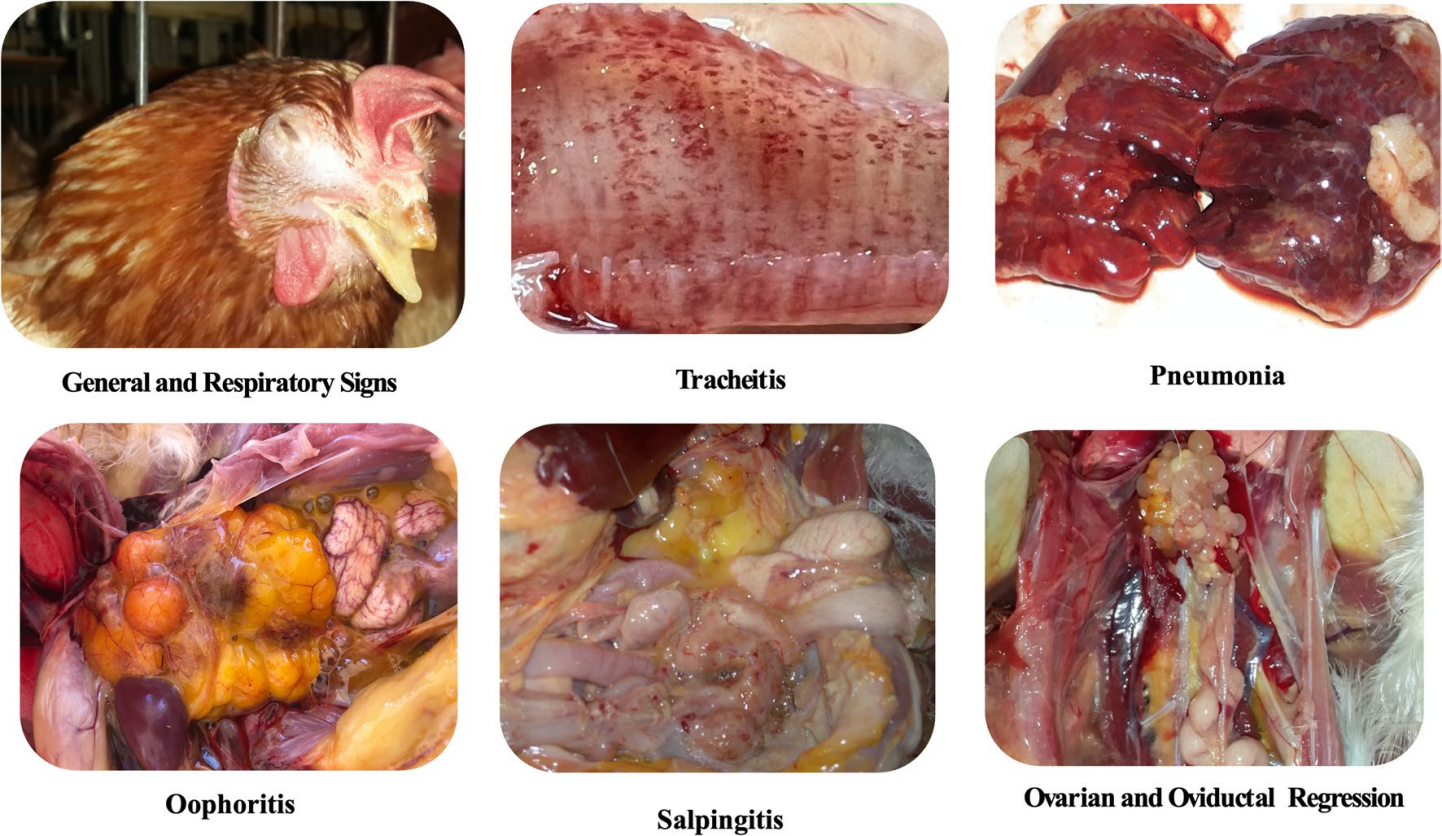
Characteristic signs and lesions of *G. anatis* infection

The post-mortem lesions of *G. anatis* are represented in Fig. 2. Respiratory, reproductive, intestinal, and septicemic lesions could be observed in infected cases with *G. anatis* (Ataei et al. 2019). Degeneration of the ovarian follicles, salpingitis, peritonitis, enteritis, and respiratory tract lesions have been associated with *G. anatis* infection (Bojesen et al. 2004). Furthermore, other *G. anatis* can induce other lesions in joints, heart, wattles, abdomen, and brain (El-Adawy et al. 2018). In the research of Neubauer et al. (2009), hemorrhagic oophoritis, damaged or malformed follicles, ovarian regression, hemorrhagic or dysfunctional oviducts, peritonitis, fibrinous perihepatitis, and pericarditis were all lesions of *G. anatis* infection in layer hens. Elewa (2021) demonstrated mild tracheitis, peritonitis, oophoritis, and salpingitis in layers with *G. anatis* infection.

Following experimental induction with *G. anatis*, layer chickens exhibited variable gross lesions such as hemorrhagic or ruptured ovarian follicles, pericarditis, multifocal hepatic necrosis, egg deformities in the oviduct, and fibrinous peritonitis (Paudel et al. 2014a). It has been noted that *G. anatis* biovar 3 caused purulent oophoritis, swollen blood vessels in the ovary, oviduct, and peritoneum, as well as purulent or fibrinous exudates in the peritoneum. Further experimental studies of *G. anatis* in chickens represented regression and deformity of ovaries, focal or diffuse salpingitis, catarrhal to purulent tracheitis, congestion of lungs, air sacculitis, pericarditis, liver congestion, and ascites (Paudel et al. 2013; Pors et al. 2016a; Abd El-Hamid et al. 2018).

*G. anatis* biovar haemolytica had been isolated from the nasal sinuses of infected cases with a nephropathogenic strain of infectious bronchitis and had respiratory signs (Franca et al. 2011). As they cause more severe peritonitis, salpingitis, and oophoritis, concurrent *E. coli* and *G. anatis* infections might worsen lesions compared to a single infection (Pors and Bojesen 2011). Besides, another study confirmed the localization of *Gallibacterium* mainly in the trachea and ovaries (Huangfu et al. 2012). *G. anatis* was isolated at a meager rate (2.7%) from pododermatitis cases in numerous layer flocks in Denmark in 2015 (Olsena et al. 2018).

## Diagnosis

**Fig. 3:**
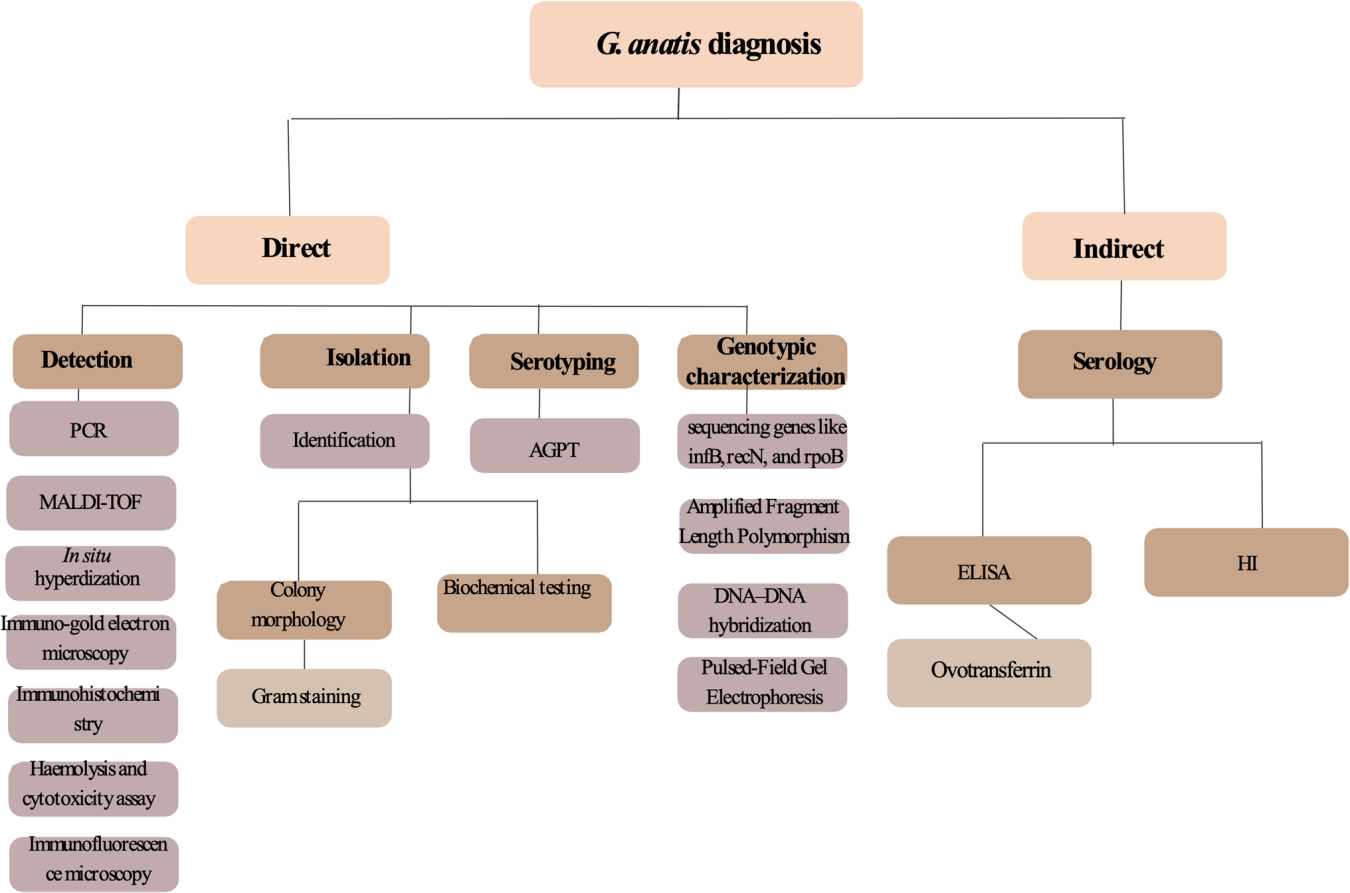
Different diagnostic tools of G. anatis

The different diagnostic tools of *G. anatis* are shown in Fig. 3.

### Isolation and phenotypic identification

The absence of pathognomonic respiratory and reproductive signs and lesions caused by *G. anatis* necessitates a specific diagnosis to confirm the infection. Isolation and phenotypic identification of *G. anatis* are the basis for diagnosing *G. anatis* infections in almost all cases (Christensen et al. 2003). However, overgrowth by other bacteria, mainly *E. coli*, significantly hinders the isolation process (Wang et al. 2016). Furthermore, phenotypic characterization techniques can produce heterogeneous, time- and labor-intensive findings. Consequently, molecular diagnostic methods that depend on identifying *16S–23S rRNA* sequences are growing (Bojesen et al. 2007b; Alispahic et al. 2012; Huangfu et al. 2012).

Species of *Gallibacterium* are usually grown at 37°C for 24–48 h on blood agar medium (with 5–10% citrated bovine blood) under facultative anaerobic/microaerophilic conditions (Christensen et al. 2003). Most *G. anatis* isolates produce a wide β-hemolytic zone with butyrous, circular, smooth, greyish, non-pigmented, and shiny colonies (El-Adawy et al. 2018). After 24–48 h of incubation, *G. anatis* develops with a modest granular deposit and weak turbidity in a nutrient broth supplemented with 5% horse serum and 3% glucose. Better bacterial growth is observed in brain heart infusion broth when 5% horse serum is added.

Biochemically, several tests are used to identify *G. anatis*, as detailed in Table 2 (Christensen et al. 2003; Bisgaard et al. 2009).

**Table 2:** Phenotypic and biochemical reactions for the identification of *G. anatis* biotypes

	*G. anatis* biovar hemolytica	*G. anatis* biovar anatis	*G. melopsittaci*	*G. trehalosifermentans*	*G. salpingitidis*	*G*. genomospecies 1	*G*. genomospecies 2	*G*. genomospecies 3	*Gallibacterium* group V
Gram stain	-	-	-	-	-	-	-	-	-
Motility	-	-	-	-	-	-	-	-	-
*β*-hemolysis	+	-	-	-	-	-	-	-	-
Growth on MacConkey’s	-/D	-	+ W	-	+	-	-	D	+
Catalase	+	+	D	D	+	D	D	D	+
Oxidase	+	+	D	D	+	D	D	D	+
ONPG	+	+	+	-	+	+	+	+	+
PNPG	+	+	-	+	-	-	-	-	-
Phosphatase	+	+	+	+	+	+	+	+	+
Nitrate reduction	+	+	+	+	+	+	+	+	+
Indole	-	-	-	-	-	-	-	D	-
Urease	-	-	-	-	D	-	-	D	+
Voges-Proskauer	-	-	-	-	-	-	-	-	-
Methyl Red	-	-	-	-	-	D	-	-	-
H_2_S	-	-	-	-	-	-	-	-	-
Citrate (Simmons)	-	-	-	-	-	-	-	-	-
Sucrose	+	+	+	+	+	+	+	+	+
Maltose	+	D	+	+	+	+	+	+	+
Ornithine decarboxylase	-	+	-	-	-	-	-	-	-
D-Arabinose	+	-	D	D	( +)	+	D	D	( +)
L-Arabinose	-	-	D	-	+	-	-	D	-
D-Arabitol	-	-	-	-	+	+	+	D	+
Xylitol	-	-	-	-	+ W	-	-	-	-
Glycerol	+	+	( +)	D	( +)	+	+	D	( +)
Mucate	-	-	D	-	+	-	-	D	+
Lactose	-	D	+ /( +)	-	( +)	-	-	D	+
D-Glucose	+	+	+	+	+	+	+	+	+
D-Ribose	+	+	+ /( +)	+ /( +)	+	+	+	D	+
D-Sorbitol	D	D	D	+	D	-	-	D	+
Trehalose	D	+	-	+	-	+	D	-	-
Raffinose	+	+	+	+	+ /( +)	+	+	D	+
Fructose	+	-	+	+	+	+	+	+	+
L-Fucose	+	-	D	+ /( +)	+	+ /( +)	D	D	+
D-Melibiose	-	-	+ /( +)	+ /( +)	D	-	-	-	+
D-Mannitol	+	+	+	+	+	D	D	D	D
D-Mannose	+	+	+	+	+	+	+	+	+
D-Xylose	+	+	D	+	+	+	+	+ /( +)	-
m-Inositol	D	D	-	-	-	-	D	-	-
Dextrin	D	-	-	-	-	( +)	+	-	-
Dulcitol	-	-	D	D	+	+	+	D	-

*ONPG* β-galactosidase, *ONPG* β-Glucosidase

Characteristics are scored as: + , all strains positive within 1–2 days; ( +), all strains positive within 14 days; − , all strains negative after 14 days; W, weakly positive; D, some strains positive, while others are negative (number of strains positive/number of strains tested)

(Christensen et al. 2003; Bisgaard et al. 2009)

Other automated identification methods like VITEK2, Phoenix100, and MALDI-TOF–MS could be used for *Gallibacterium* identification (Alispahic et al. 2012; Allahghadry et al. 2021).

### Matrix-assisted laser desorption/ionization time-of-flight mass spectrometry (MALDI-TOF–MS)

MALDI-TOF–MS is an effective technique for identifying *G. anatis* biomarkers (El-Adawy et al. 2018). This test has an impressive prospective for a routine laboratory diagnosis of the bacteria as it is rapid, can proceed with many samples concurrently, and needs small sample sizes. This test identified one clonal lineage of *G. anatis* in various flocks (Alispahic et al. 2011, 2012; Allahghadry et al. 2021). The genetic diversity of *Gallibacterium* isolated from 13 farms with various biosecurity measures and management techniques was examined using phylogenetic analysis of incomplete *rpo*B sequences and biotyping using MALDI-TOF–MS. The findings showed significant variability among isolates from farms with poor biosecurity standards and those with greater biosecurity standards (more closely linked and grouped). Low biosecurity standards allow viruses to spread horizontally, and gene transfer creates genetic diversity (Lozica et al. 2020).

### Molecular detection

Molecular diagnosis of *G. anatis* has been recently developed, being fast, easy, highly specific, sensitive, and reliable (Ataei et al. 2017). Molecular assays such as polymerase chain reaction (PCR) are confirmatory to both phenotypically detected *G. anatis* strains and the negative samples following initial isolation (Bisgaard et al. 2009; Elewa 2021). Primers, including *16S* to *23S rRNA* internal transcribed spacer sequence, are specific to *Gallibacterium* (Bojesen et al. 2007a) and used for its differentiation from other *Pasteurellaceae* generas (Christensen et al. 2003). In addition, the primer 1133*fga*l is specific for *Gallibacterium*, not for other members of *Pasteurellaceae*. The *23S rRNA* gene sequence primer 114*r* is utilized as a reverse primer (Lane 1991). Three specific amplicons of around 789, 985, and 1032 base pair can be identified in *G. anatis* infection (Neubauer et al. 2009; Singh, 2016; Singh et al. 2016; Ataei et al. 2017; Wang et al. 2018).

When phenotypic identification of *Gallibacterium* is challenging, classification based on the gene sequence of the DNA-dependent RNA-polymerase (rpoB) subunit might be utilized (Korczak et al. 2004; Christensen et al. 2007b). The *gtx*A-encoding gene, the fimbrial gene, and the gyrase subunit B gene may all be found using the PCR approach (Sorour et al. 2015; Krishnegowda et al. 2020).

Real-time quantitative PCR (qPCR) is used as a species-specific identification and quantifying method for *G. anatis* (Huangfu et al. 2012; Wang et al. 2016, 2018). The protein gyrase subunit B gene is essential for the function of the DNA replication enzyme because it encodes the ATPase domain of this enzyme. The qPCR is more rapid, highly specific, sensitive, reproducible, and cost-effective, and it needs a lower DNA concentration than conventional PCR or phenotypic characterization methods (Wang et al. 2016; Huangfu et al. 2012).

Real-time loop-mediated isothermal amplification PCR assay has recently been used to detect *G. anatis*. This assay targets the *sod*A gene. It has been described as sensitive, rapid, and specific for *G. anatis* identification. Moreover, it is quicker and cheaper than quantitative PCR (Stępień-Pyśniak et al. 2018).

Genotypic characterization methods of *G. anatis* have been used, such as DNA–DNA hybridization, pulsed-field gel electrophoresis, amplified fragment length polymorphism, and sequencing genes like *inf*B, *rec*N, and *rpo*B (Christensen et al. 2003; Bisgaard et al. 2009). Moreover, Allahghadry et al. (2021) detected 84 (70%) of *G. anatis* out of 120 layers and broiler chicken tracheal samples, and after genotyping by pulsed-field gel electrophoresis and genome sequencing revealed a total of 24 pulsotypes for 71 *G. anatis* strains (87% similarity level) and 7 genome clusters including 21 strains (97% similarity level), respectively.

### Other diagnostic techniques

#### Agar gel precipitation test

Galaz-Luna et al. (2009) used an agar gel precipitation test for serotyping and cross-reactivity and found no relationship between biovar and serovar among the species of *Gallibacterium*.

#### Enzyme-linked immunosorbent assay

Serological testing is helpful for flock monitoring or diagnosing *Gallibacterium* infection. Wang (2012) developed an indirect enzyme-linked immunosorbent assay (ELISA) to recognize all antibodies against *G. anatis* in chickens. The response curve was established when the optical density 450 nm values varied along with the time. The antibody level peaked in 2-month pi, but for a short period, and then gradually dropped. Nowadays, ovotransferrin can be detected in chicken serum using ELISA as an acute phase protein marker in experimental *G. anatis* infections (Roy et al. 2014). Acute-phase proteins are good markers for diagnosis and prognosis.

#### Haemagglutination test

As *G. anatis* is a haemagglutinating bacterium for avian and mammalian RBCs [chickens, turkeys, pigeons, quails, ducks, Harris’s hawks (*Parabuteo unicinctus*), house finches (*Carpodacus mexicanus*), cows, sheep, horses, dogs, rabbits, pigs, and humans (groups A, B, AB, and O; Rh +)]; thus, haemagglutination test is used for the rapid detection of the organism using microdilution method or microtiter plates (Zepeda et al. 2009; Montes-Garcia et al. 2016).

#### Hemolysis and cytotoxicity assay

The hemolysis assay of *G. anatis* can be adopted using washed bovine blood RBCs and detected using ELISA, while the cytotoxicity assay can be applied on HD11 cells in 96-well tissue culture plates (Kristensen et al. 2010). The GtxA is the cause of cytotoxicity and hemolysis (Kristensen et al. 2011).

#### Immunofluorescence microscopy

The culture of *G. anatis* is fixed with paraformaldehyde on glass slides and labeled with anti-fimbriae immune serum. The conjugated goat anti-rabbit secondary antibodies are added, the slides are mounted, and the images are captured using laser scanning microscopy (Bager et al. 2013b).

#### In situ* hybridization*

The *16S rRNA* of *Gallibacterium* is the target of an in situ hybridization probe dyed cyanine. The pathogenic changes in the spleen and liver tissues of experimentally infected hens have been studied using this hybridization approach (Bojesen et al. 2003a, 2004). This technique is vital for the detection of *G. anatis* pathogenies and dissemination.

#### Immuno-gold electron microscopy

This method is processed as an immunofluorescence assay, but the secondary antibodies are gold particles on nickel grids coated with Formvar carbon (Bager et al. 2013b). Electron microscopy is used for the detection of *G. anatis*.

#### Histopathology and immunohistochemistry

Several studies reported the histopathological examinations. Following the intravenous or intraperitoneal inoculation of virulent *G. anatis* strains in normal or immunosuppressed 15-weeks-old brown laying chickens, the liver lesions in the intravenously infected birds included basophilic aggregates (*Gallibacterium* microcolonies) bordered by necrotic hepatocytes, non-identifiable necrotic cells, proteinaceous fluid, and eosinophilic and basophilic aggregates in the ellipsoids in the spleens. After 12 and 24 h pi, the histopathological changes included forming multinucleated giant cells around some of the ellipsoid lesions in the spleen and liver. At the same time, the intraperitoneally infected chickens with a normal immune status showed diffuse purulent peritonitis and fibrinous perihepatitis 12 h pi (Bojesen et al. 2004; Zepeda et al. 2010). A combined infection of *G. anatis* and other pathogens was studied, and the results indicated changes in the lung, trachea, oviduct, and ovary regarding hyperemia, swelling, epithelium cell degeneration, and necrosis in the mucosa of the respiratory tract. The mucosa of the oviduct presented several neutrophilic granulocytes and ovarian cysts (Hui-min et al. 2009). The different histopathological severity degrees were due to the differences in virulence among the used *G. anatis* biovar hemolytica in chickens (Zepeda et al. 2010; Abd El-Hamid et al. 2016; Mataried 2016).

Immunochemistry was used to detect the ability of *G. anatis* isolates to adhere to or invade the chicken oviduct epithelial cells using polyclonal antibodies. The results of this assay revealed that *G. anatis* could attach epithelial cells without invasion (Zhang et al. 2017).

## Prevention and control strategies

Adopting effective biosecurity measures, “all in-all out,” in poultry farms prevents the horizontal transmission of *G. anatis*, the gene transfer, and consequently, the heterogeneity or the diversity of this pathogen (Lozica et al. 2020). Treatment of a mixed infection with other bacteria using a specific drug, controlling of other immunosuppressive agents, and amelioration of stress conditions are the must to prevent or control *G. anatis* infection (Mataried 2016; Paudel et al. 2017a, b; Abd El-Hamid et al. 2018). Eggs or hatchery cleanliness is crucial to prevent trans-eggshell transmission caused by fecal contamination with *G. anatis* (Wang et al. 2018).

### Antibiotic treatment

The administration of effective antimicrobials is needed to treat *G. anatis* infections. As of now, the antigenic diversity among *G. anatis* strains and MDR causes treatment failure and hinders vaccination-based prophylaxis (Bojesen et al. 2003b, 2007c; Christensen et al. 2003; Bojesen et al. 2011a, b; Johnson et al. 2013; Jones et al. 2013; Chavez et al. 2017b; Hess et al. 2019). In this regard, Elbestawy (2014) detected absolute resistance of 20 isolates of *G. anatis* to oxytetracycline and lincospectin and owed this resistance to the extensive exposure of layer chicken flocks in Egypt to long courses of these antibiotics in feed as a rational prophylactic program. A similar resistant pattern was obtained in Morocco by Nassik et al. (2019), who found that all *G. anatis* strains were resistant to ampicillin, erythromycin, oxytetracycline, and sulfamethoxazole-trimethoprim.

Besides, Bojesen et al. (2011b) reported that field strains of *G. anatis* were resistant to sulfamethoxazole and tetracycline at rates of 97% and 92%, respectively, while reference strains were resistant to the previous antimicrobials at rates of 42% and 67%, respectively. Alarmingly, MDR has markedly increased worldwide in the last decade, which is deemed a public health threat. Numerous recent epidemiological studies established the occurrence of extensive drug resistance and MDR bacterial pathogens from distinct origins, including humans, fish, food products, and poultry (Abd El-Ghany, 2021; Algammal et al. 2021b, c; Hetta et al. 2021; Algammal et al. 2022b).

The antimicrobial resistance (AMR) of *G. anatis* strains isolated from chickens in different localities were studied using PCR and cloning and sequencing of plasmid-mediated resistant gene of streptomycin, sulfamethoxazole, and fluoroquinolones (Bojesen et al. 2011a, b; Guo, 2011; Gao, 2012; Monita et al. 2011). The antimicrobial susceptibility for 84 chicken isolates of *G. anatis* from broiler and breeder flocks was studied in the USA compared to *P. multocida* isolates. Amoxicillin, neomycin, and sulfonamide-trimethoprim resistance levels were superior in *G. anatis* than in *P. multocida* (Jones et al. 2013). Resistance to tetracycline could be due to the genes *tet*B, *tet*H, and *tet*L, which have been detected in strains of *G. anatis* (Abd El Tawab et al. 2018).

El-Adawy et al. (2018) reported that 93% of the field *G. anatis* strains were resistant to sulfamethoxine, 93% to spectinomycin, 87% to tylosin, and 80% to oxytetracycline, but they were sensitive to apramycin, florfenicol, and neomycin. Chavez et al. (2017b) found resistance of *G. anatis* isolates to penicillin, tylosin, lincomycin, ampicillin, enrofloxacin, oxytetracycline, norfloxacin, and cephalexin, but sensitivity to ceftiofur (73%) and florfenicol (68%). Moreover, Elbestawy et al. (2018) revealed antibiotic sensitivity differences among *G. anatis* isolates obtained during 2013 and 2015. The highest antibiotic sensitivity results for 2013 *G. anatis* isolates were to florfenicol, ciprofloxacin, and norfloxacin, respectively, while those isolates of 2015 were susceptible to cefotaxime, followed by florfenicol, norfloxacin, and ciprofloxacin. In Nigeria, the sensitivity of non-hemolytic and hemolytic biovars of *G. anatis* isolated from Muscovy ducks was high to cefotaxime, ciprofloxacin, doxycycline, and florfenicol (Lawal et al. 2017). In layer flocks, 96% of *G. anatis* isolates showed MDR to tylosin (95%), tetracycline (89%), nalidixic acid or sulfamethoxazole (77%), and enrofloxacin (58%) (Hess et al. 2019).

In Egypt, a recent study by Elewa (2021) showed that all the isolated layer strains of *G. anatis* were resistant to kanamycin and neomycin (98%), penicillin and ampicillin (96%), oxytetracycline (95%), and sulfamethoxazole-trimethoprim and norfloxacin (27.5%), but susceptible to erythromycin and azithromycin (96%), florfenicol (90%), sulphamethaxole trimethoprim (57.3%), and norfloxacin (44%). Another recent Romanian study revealed that all *G. anatis* strains showed a resistant profile to tetracyclines, fluoroquinolones, ampicillin, trimethoprim, nalidixic acid, and clindamycin (Sorescu et al. 2021). Furthermore, Allahghadry et al. (2021) showed a lower sensitivity of 2 *G. anatis* strains to tetracycline (76.2%) and enrofloxacin (90.5%), suggesting the widespread occurrence of MDR *G. anatis* isolates and announced calls for non-antibiotic prophylactics.

Owing to the emergence of widespread MDR strains of *G. anatis*, traditional antimicrobials are not suggested for treating *G. anatis* infection. In the study by Prasai et al. (2017), feed supplementation with zeolites (aluminosilicate minerals) showed an effective reduction of *G. anatis* in chickens. In addition, Zhang et al. (2019) reported that specific chicken egg yolk IgY produced against recombinant N-terminal of GtxA revealed significant protection against *G. anatis* infection and decreased the severity of the lesion in the liver and intestine. Moreover, a recent study by Zhang et al. (2021) showed that a strain of *Leuconostoc mesenteroides* (QZ1178) exerts potential in vitro antibacterial effects against *G. anatis* through lactic acid formation.

### Vaccines

Prior to now, the effectiveness of conventional vaccinations in protection against *G. anatis* infection was limited due to the antigenic diversity reported by Krishnegowda et al. (2020). A bacterin including 3 different biovars accounting for 95% of the outbreaks in Mexico has been developed by Boehringer Ingelheim, Mexico. The serological response was monitored from week 9 to week 40 using a custom-made ELISA test for *G. anatis*. The results indicated that the birds vaccinated with *G. anatis* bacterin were high in antibody titers and egg production (Gonzalez et al. 2003; Vazquez et al. 2003). The prepared autogenous bacterin was very effective in protecting against clinical disease and decreasing egg production and performance in layers (Castellanos et al. 2007) and in broiler breeder flocks (Calderon and Thomas 2009).

The vaccination potential of many immunogenic antigens such as GtxA, OMVs, fimbriae, capsules, elongation factor Tu, CRISPR, and recombinant proteins (GtxA and fimbriae) has been studied (Bager et al. 2013a, 2014; Pedersen et al. 2015; Pors et al. 2016a, b). Moreover, the efficacy of various immunogenic proteins such as GtxA, fimbriae, *Gab_1309*, and *Gab_2348* for epitope recognition and prediction was investigated. The results revealed that these immunogenic proteins could stimulate the immune response (Ataei et al. 2019). Vaccination of layer chickens with OMVs of *G. anatis* decreased the severity of lesions and increased the serum titers of specific immunoglobulin (Y) (Pors et al. 2016b). Immunization with OMVs and the fimbrial protein of *G. anatis* could protect against the disease (Persson et al. 2018). A killed bivalent commercial vaccine (bacterin), including *A. paragallinarum* and *G. anatis* antigens, can induce complete protection in immunized chickens (Paudel et al. 2017a,b). Finally, Allahghadry et al. (2021) detected three major immunogen genes, *gtx*A, *Gab_1309*, and *Gab_2312*, in the examined *G. anatis* isolates, which possibly exemplify efficient vaccine targets, and the authors suggested the use of the *gtx*A gene (the most conserved sequence in all *G. anatis* strains) for the future vaccine development purposes (recombinant vaccines).

In conclusion, periodic studies regarding *Gallibacterium* infections should be applied to control their economic losses and improve poultry production. These studies should investigate the current epidemiological situation of the disease in the locality, the sources of infection in terms of public health, the antibiogram profile of the isolated strains, and the development of future vaccines. In addition, it is crucial to discuss the precise biology and pathophysiology of *Gallibacterium* infection and how this bacterium switches from being a normal inhabitant flora to a pathogen producing a disease condition in the host.

## Data Availability

The datasets generated during the current study are available from the corresponding author upon reasonable request.
